# Hearing Loss, Brain Structure, Cognition, and Dementia Risk in the Framingham Heart Study

**DOI:** 10.1001/jamanetworkopen.2025.39209

**Published:** 2025-11-05

**Authors:** Francis B. Kolo, Sophia Lu, Alexa S. Beiser, Lily Francis, Debora Melo van Lent, Monica Gireud-Goss, Jayandra J. Himali, Saptaparni Ghosh, Shu Jie Ting, Ayantika Banerjee, Timothy L. Kowalczyk, Jared M. Zucker, Nancy Heard-Costa, Christopher G. Wood, Pauline Maillard, Evan M. Fletcher, Charles S. DeCarli, D. Bradley Welling, Sharon G. Kujawa, Sudha Seshadri

**Affiliations:** 1 Glenn Biggs Institute for Alzheimer’s and Neurodegenerative Diseases, The University of Texas Health Science Center at San Antonio; 2 Department of Biostatistics, Boston University School of Public Health, Boston, Massachusetts; 3 Department of Neurology, Boston University Chobanian and Avedisian School of Medicine, Boston, Massachusetts; 4 Framingham Heart Study, Framingham, Massachusetts; 5 Sarah Cannon Research Institute, Cambridge, Massachusetts; 6 Harvard Medical School, Department of Otolaryngology Head and Neck Surgery, Massachusetts Eye and Ear Infirmary and Massachusetts General Hospital Boston; 7 Department of Neurology and Imaging of Dementia and Aging Laboratory, University of California Davis, Sacramento

## Abstract

**Importance:**

Age-related hearing loss is a potentially modifiable risk factor for cognitive impairment and dementia.

**Objective:**

To investigate the association of hearing loss with brain structure changes, cognitive function, and incident dementia.

**Design, Setting, and Participants:**

This cohort study included Framingham Heart Study Offspring Study participants attending their sixth quadrennial examination (1995-1998) divided into 2 partially overlapping samples. Sample 1 participants underwent brain magnetic resonance imaging and cognitive assessment at their seventh and eighth quadrennial examinations. Sample 2 included participants aged 60 years or older at pure tone average (PTA) analysis and subsequently followed up for incident dementia. The data analysis was performed between January 12, 2024, and August 24, 2025.

**Exposure:**

Hearing loss.

**Main Outcomes and Measures:**

The main outcomes were changes in brain volume on MRI, cognitive performance on neuropsychological testing, and incident all-cause dementia. Multivariable linear regressions were used to compare PTA and hearing loss categories with neuropsychological measures and total cerebral, hippocampal, and white matter hyperintensity brain volumes. Cox proportional hazards regression was used to examine longitudinal associations between PTA and hearing loss categories and incident dementia.

**Results:**

Sample 1 included 1656 participants (mean [range] age, 58.1 [29.7-85.6] years; 903 female [54.5%]), and sample 2 included 935 participants (mean [range] age, 67.6 [60.0-85.6] years; 518 female [55.4%]). Participants with mild or greater hearing loss, compared with those with no or only slight hearing loss, had significantly smaller brain volumes (β [SE], –4.10 [1.76]) and declines in executive function (β [SE], −0.04 [0.01]). Participants with at least slight hearing loss, compared with those with no hearing loss, had significant increases in white matter hyperintensity volume (β [SE], 0.02 [0.01]) and a higher risk of developing all-cause dementia (hazard ratio, 1.71; 95% CI, 1.01-2.90) over 15 years of follow-up, particularly among those with at least 1 apolipoprotein E ε4 allele (hazard ratio, 2.86; 95% CI, 1.12-7.28).

**Conclusions and Relevance:**

This cohort study found that midlife hearing loss was associated with smaller brain volumes, accelerated worsening in executive function, and an increased risk of dementia, suggesting that it may be an early marker of brain aging and dementia risk.

## Key Points

**Question:**

Is midlife hearing loss associated with markers of brain aging, cognitive decline, and risk of developing dementia?

**Findings:**

In this cohort study of 2178 Framingham Heart Study participants, mild or greater hearing loss was associated with smaller brain volume, greater white matter abnormalities, and declines in executive function over time. Any hearing loss was associated with a 71% increased risk of developing dementia, especially in persons with at least 1 apolipoprotein E ε4 allele, with hearing aid use mitigating these risks.

**Meaning:**

These findings suggest that midlife hearing loss may be an early marker of brain aging and dementia risk, particularly among persons with at least 1 apolipoprotein E ε4 allele.

## Teaser

This cohort study investigates whether midlife hearing loss is associated with brain structure changes, cognitive function, and incident dementia.

## Introduction

Age-related hearing loss may be a modifiable risk factor for cognitive decline and dementia^1^ and is becoming more prevalent as the global population ages.^2^ The prevalence of hearing loss (audiometric threshold elevations) in persons older than 65 years ranges from 29% to 47% worldwide.^3,4,5,6^ *The Lancet* International Commission on Dementia Prevention and Care^7^ identified hearing loss as a major risk factor for dementia. Several population-based cohort studies, such as the Beaver Dam study,^8^ the Baltimore Longitudinal Study of Aging,^9^ and the Hispanic Community Health Study,^10^ have reported associations of hearing loss with poorer cognition. Other studies have associated mild or greater hearing loss with smaller brain volumes on magnetic resonance imaging (MRI) but did not evaluate cognitive function.^11,12,13^ A few cross-sectional studies with small sample sizes have associated mild or greater hearing loss^14,15^ with both cognitive function using neuropsychological test performance and MRI.^16,17^ Data from the Baltimore Longitudinal Study of Aging^18^; the Health, Aging, and Body Composition study^19^; and the National Alzheimer’s Coordinating Center have associated hearing loss with risk of incident dementia^20,21^; however, they did not examine MRI measures.

In this study, we aimed to examine cross-sectionally and longitudinally the association of hearing loss with a range of brain-related outcomes, including neuropsychological and MRI measures, and incident dementia in a single study population, the Framingham Heart Study (FHS) Offspring Study. We evaluated the modification of this association by the presence or absence of an apolipoprotein E (*APOE*) ε4 allele. Additionally, we examined the incremental estimated value of adding hearing loss information to demographic, genetic, and vascular risk factors in estimating the risk of dementia.

## Methods

### Study Population

This cohort study used data from the FHS, an ongoing, community-based, 3-generational, prospective cohort study located in Framingham, Massachusetts.^22^ Approval for this FHS research was obtained from the Institutional Review Board of Boston University School of Medicine. Written informed consent was obtained from all participants or their legally authorized representative before each examination. This study adhered to the Strengthening the Reporting of Observational Studies in Epidemiology (STROBE) reporting guideline.

In 1971, the FHS Offspring Study cohort (generation 2)^23^ enrolled 5124 participants who had been studied over 10 examination cycles approximately once every 4 years. For our analyses, we selected 2 partially overlapping samples, both derived from 3532 persons who attended their sixth offspring examination (1995-1998) and 2178 persons who completed a hearing assessment.

### Examination Procedures

A certified audiologist (C.G.W.) conducted pure tone threshold testing in a sound-attenuating test booth meeting American National Standard Institute section 3.6-1969, subsection 2.1.1 (published in 1969), specifications.^24,25,26^ Air conduction thresholds were obtained at octave frequencies of 0.5, 1.0, 2.0, 4.0, and 8.0 kHz. A hearing history was obtained from each participant at the time of audiometry. The questionnaire (available upon request) asked about the presence of hearing loss (subjective hearing loss); Ménière disease; tinnitus; dizzy spells; otosclerosis; head injury; any acute illness, such as meningitis or otitis, that could result in hearing loss; ear surgery; family history of hearing loss; exposure to noise; use of ototoxic medicines; and hearing aid use. Otoscopy and tympanometry were conducted before audiometry to evaluate the integrity of the ear canal and ear drum and exclude persons with possible middle ear disease, either past or present.

### Exposure

Hearing loss as the exposure variable was analyzed using pure tone averages (PTAs) across frequencies of 0.5, 1.0, 2.0, and 4.0 kHz and calculated separately for each ear. We analyzed PTAs of the better ear (the ear with the lower PTA threshold). We considered the continuous PTA, natural log transformed to normalize its skewed distribution, and defined hearing loss based on the modified National Health and Aging Trends Study thresholds categorization, in which the degree of hearing loss is categorized as normal hearing (range, 0-16 decibels hearing loss [dB HL]), slight (range, 16-26 dB HL), mild (range, 26-40 dB HL), and moderate or greater (>40 dB HL).

### Outcomes

Persons who attended the seventh (1998-2001) and eighth (2005-2008) quadrennial examinations were invited to participate in a callback study, which was a follow-up evaluation in which FHS participants who attended a 4-hour quadrennial examination were invited back for more detailed assessments. Such assessments included brain MRI scans and/or neuropsychological testing that could not be completed at the baseline visit either due to the length of time required (neuropsychological testing) or the offsite location of necessary equipment (MRI scanner). Both the MRI and neuropsychological testing were typically done on the same day.

### Cognitive Assessment With Neuropsychological Tests

We assessed cognitive performance and decline using a battery of validated neuropsychological tests.^27^ Each test examined a specific cognitive domain. The Visual Reproductions Immediate and Delayed Recall (VR-i and VR-d) and the Logical Memory Immediate and Delayed Recall (LM-i and LM-d) tests of the Wechsler Memory Scale measured visual and verbal memory, respectively.^28^ The Similarities test, as part of the Wechsler Adult Intelligence Scale, measured verbal comprehension and reasoning.^29^ Trail Making Test (TMT) part A (TMT-A) and part B (TMT-B) measured visual search skills and motor speed in tests of greater complexity,^28,30^ whereas TMT-B minus TMT-A scores assessed participants’ cognitive flexibility independent of manual dexterity.^28,30,31^ The Hooper Visual Organization Test (HVOT) assessed visuoperceptual skills.^28^ The Paired Associates Learning–Total Score is a subset of the Wechsler Memory Scale and measures new learning and, again, immediate and delayed recall.^27,32,33^ Higher scores across all cognitive end points indicate superior performance, except for the TMT-A and TMT-B, in which higher scores indicate greater time taken for task completion; hence, we reversed the sign for the latter 2 tests to have a directionality consistent with the other neuropsychological variables, such that higher scores always indicate better cognition. We also natural log transformed HVOT and TMT-B minus TMT-A scores to normalize their skewed distribution. We created a global cognition measure derived using principal component analysis (ie, forcing a single principal component solution [principal components 1-6v (PC1-6v)]). This measure produced a composite global cognitive score based on the participant’s performance on 6 items in the cognitive battery.^28^ Briefly, task scores were standardized (ie, *z* scores were created) and summed according to their weighting to the overall cognitive factor^34^ to produce a composite measure of cognitive function reflecting general cognitive ability. The composite score (PC1-6v) combined weighted loadings for (LM-i + LM-d), (VR-i + VR-d), HVOT, Paired Associates Learning–Total Score, Similarities test, and TMT-B. We calculated the annualized change in these variables between the examination 7 (1998-2004) and examination 8 callback assessments (2004-2011). We defined a priori 3 primary cognitive outcomes: TMT-B minus TMT-A, LM-i + LM-d, and PC1-6v, covering executive, memory, and global cognition, respectively.

### Brain MRI

Brain MRI was obtained using either a MAGNETOM Expert or Avanto machine (Siemens Healthineers) (1 T at examination 7 and 1.5 T at examination 8) and included high-resolution 3-dimensional T1-weighted magnetization-prepared rapid acquisition gradient echo images used to derive the measures we describe. Analysis of MRI images was completed by a team of skilled brain imaging analysts (P.M., E.M.F., and C.S.D.) working with neurologists and neuroradiologists.^35^ Quantification included the automatic removal of nonbrain elements from the 3-dimensional T1 image volume using a robust and accurate convolutional neural network method relatively insensitive to machine type or field strength.^36^ Intracranial volume was defined as the total space within the cranial vault along the inner surface of the dura lining the skull and above the tentorium. Image intensity correction was used to remove B1 inhomogeneity effects,^37^ and images were further segmented into 4 tissue types (gray, white, cerebrospinal fluid, and white matter hyperintensities) using previously published methods.^36,37,38,39^ Additionally, hippocampal volumes were measured using an atlas-based diffeomorphic approach^40^ with the minor modification of label refinement. We generated the following MRI measures: total cerebral brain (parenchymal) volume (TCBV); hippocampal volume (HPV); and white matter hyperintensity volume (WMHV), which quantifies the total volume of white matter hyperintensities in the brain. The WMHV was natural log transformed to normalize its skewed distribution. Annualized changes in TCBV, HPV, and WMHV from examinations 7 to 8 were calculated. Of these measures, we defined TCBV and WMHV as our primary outcomes.

### Incident Dementia

In sample 2, incident all-cause dementia and dementia of the Alzheimer type were ascertained through continuous surveillance.^41^ The FHS Offspring Study cohort participants have been screened at each examination visit since 1991 using the Mini-Mental State Examination. Participants flagged for possible cognitive impairment or dementia, as well as participants who developed symptoms between quadrennial examinations and were identified at an annual health history update or through self-, family, or physician referral, were subsequently evaluated by a neurologist and neuropsychologist. Participants suspected of having dementia had their complete study and outside records referred for a consensus dementia review.

A diagnosis of dementia was based on a review of all available neurologic examination records; neuropsychological assessments; neuroimaging investigations; hospital, nursing home, and outpatient clinic records; family interviews; and autopsy results (if available) by a dementia review committee that included a minimum of 1 neuropsychologist and 1 neurologist. Participants with suspected cognitive impairment who did not meet the diagnostic criteria for dementia underwent regular neuropsychological and neurologic assessments between the scheduled examinations. If a participant died or did not attend further follow-up examinations, the dementia review committee reviewed medical records up to the time of death or loss to follow-up to ascertain whether the participant may have developed interval cognitive impairment or dementia prior to the censoring date.

All-cause dementia was diagnosed using criteria from the *Diagnostic and Statistical Manual of Mental Disorders, Fourth Edition*,^42^ and dementia of the Alzheimer type was diagnosed based on the criteria of the National Institute of Neurological Disorders and Stroke and the Alzheimer’s Disease and Related Disorders Association for possible or probable Alzheimer disease.^43^ We subsequently reviewed and updated diagnoses to ensure conformity with the National Institute on Aging–Alzheimer’s Association criteria for possible, probable, and definite Alzheimer dementia,^44^ and these diagnoses are used in our study. For this updated categorization, when amyloid and tau status were known based on positron emission tomography imaging obtained for clinical or research purposes, or through a lumbar puncture conducted by the participant’s treating physician, this information was considered in the diagnostic categorization. Similarly, if autopsy data were available, these were incorporated into the final diagnosis (125 participants). The date of onset of clinical dementia and diagnosis of dementia subtype were also ascertained at this dementia review committee meeting.

### Covariates

Covariates adjusted for in this study included sex, age, and education level categorized as a 4-class variable: no high school degree, high school degree, some college, and at least 1 college degree. The cohort included 100% non-Hispanic White participants. In secondary analyses we adjusted for systolic blood pressure (SBP), presence or absence of type 2 diabetes (T2D), and current smoking status at examination 6. Type 2 diabetes was defined as having a fasting blood glucose level at or above 126 mg/dL (7 mmol/L) or being on medication for diabetes. *APOE* ε4 allele genotyping was determined as previously described.^45,46^

### Statistical Analysis

#### Cognition and Brain Structure

We performed cognition and brain structure analyses in sample 1. We defined cognitive testing scores at examination 7 as the baseline and divided the difference in scores on each cognitive measure between examinations 7 and 8 by the time interval between these examinations in years to arrive at an annualized rate of change. We compared PTA as a continuous variable and hearing loss categories (including subjective hearing loss) with neuropsychological measures (baseline and change) using multivariable linear regressions adjusted for age, age squared (to allow for a nonlinear association), sex, education, and the time between hearing assessment and the baseline neuropsychological evaluation. Similarly, we conducted multivariable linear regressions to assess the associations between PTA and hearing loss categories and TCBV, HPV, and WMHV (baseline and change), adjusted for age, age squared, sex, head size, and time between hearing assessment and baseline MRI.

#### Incident Dementia

We used Cox proportional hazards regression to examine the longitudinal associations between continuous measures of PTA and hearing loss categories and incident all-cause dementia, as well as Alzheimer disease. The proportional hazards assumption was confirmed using weighted Schoenfeld residuals. We present adjusted hazard ratios (HRs) accompanied by 95% CIs, adjusting for age, sex, education, and *APOE* ε4. We used Fine-Gray methods to adjust for the competing risk of death.

#### Secondary Analyses of Neuropsychological Testing, MRI, and Dementia Outcomes

We conducted additional analyses adjusting for key vascular and lifestyle-related covariates, including SBP, T2D, and current smoking status. We also examined effect modification by median age and sex and for the dementia analyses, presence or absence of an *APOE* ε4 allele by including interaction terms in each primary model. For significant interactions, stratified analyses were conducted.

All statistical analyses were conducted between January 12, 2024, and August 24, 2025, using SAS, version 9.4 (SAS Institute Inc), and statistical significance was set at *P* < .10 for interaction analyses and *P* < .05 for all other analyses. The threshold for statistical significance was 2-sided.

## Results

**Figure 1.**
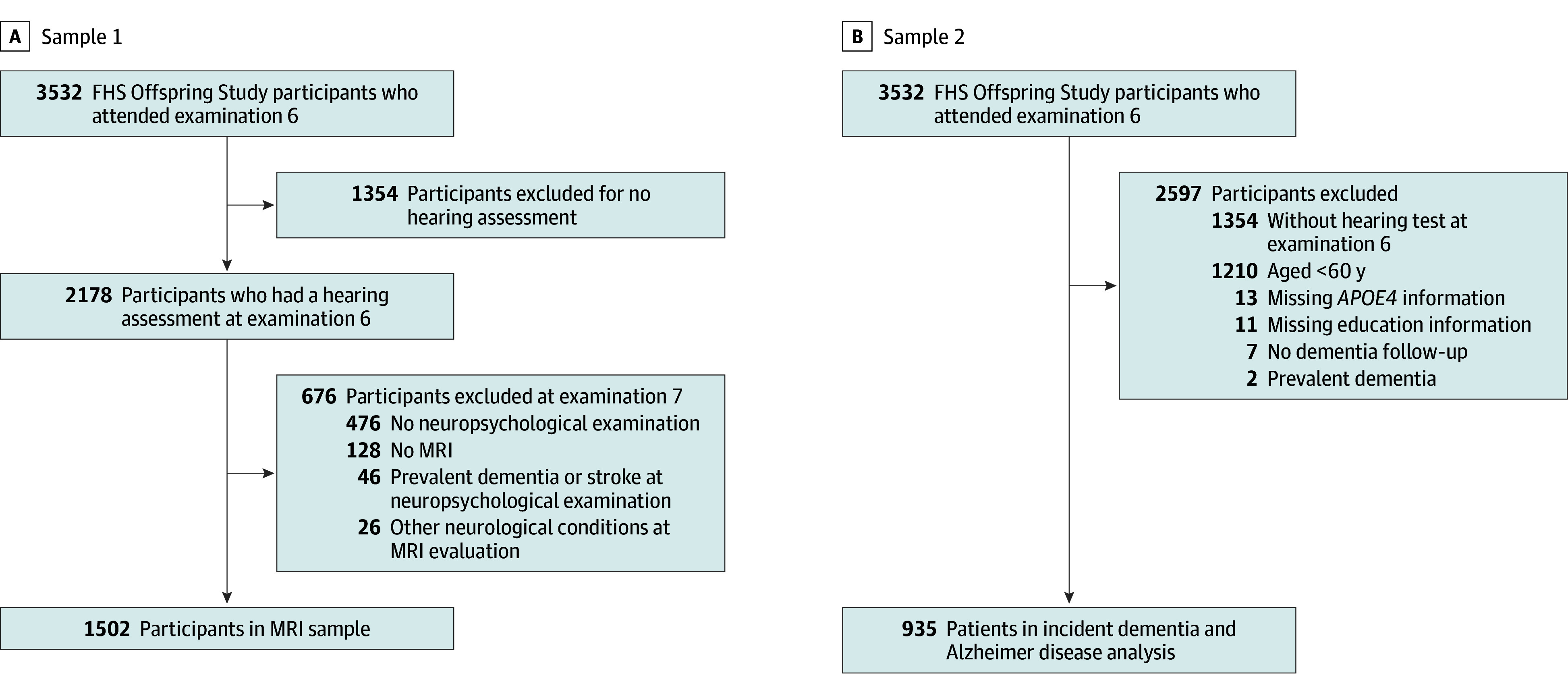
Sample 1 and Sample 2 Flow Diagrams A, Sample selection for the examination of the association between hearing loss and brain structure (magnetic resonance imaging [MRI]) and cognition (neuropsychological testing) at the Framingham Heart Study (FHS) seventh examination. After excluding 476 participants without a neuropsychological test and 46 with prevalent dementia or stroke, the neuropsychological sample in this analysis was 1656 participants. B, Association between hearing loss and incident dementia in participants older than 60 years at examination 6. *APOE* indicates apolipoprotein E.

For the cognitive and MRI analyses (sample 1), we began with 1702 FHS Offspring Study participants who also had cognitive testing done as a callback assessment after their seventh examination (1998-2001) and excluded those with dementia or stroke at that examination, retaining 1656 participants (mean [range] age, 58.1 [29.7-85.6] years; 903 female [54.5%] and 753 male [45.5%]) and, thus, permitting us to investigate the impact of midlife hearing loss on the brain (Figure 1A). Of these individuals, 1528 additionally underwent brain MRI, but we excluded 26 with unrelated brain MRI abnormalities (eg, tumor, contusion) that would affect volumetric analyses. For the incident dementia analyses (Figure 1B), among the 2178 individuals with hearing assessments at examination 6, 968 were aged 60 years or older at hearing assessment; others were excluded for having too low a risk of developing dementia in the subsequent decade. After excluding individuals with prevalent dementia and those missing information on the *APOE *ε4 genotype, educational status, or cognitive change after their sixth examination, we included 935 (mean [range] age, 67.6 [60.0-85.6] years; 518 female [55.4%] and 417 male [44.6%]) in sample 2. Sample 1 and 2 demographic characteristics are shown in Table 1.

**Table 1.** Baseline Characteristics of the FHS Offspring Study Participants Undergoing Neuropsychological Testing and Those Free of Incident Dementia at Examination 7

Characteristic	Participants No./total No. (%)	
	Neuropsychological testing sample	Incident dementia sample
Age, mean (range), y	58.1 (29.7-85.6)	67.6 (60.0-85.6)
Sex		
Female	903/1656 (54.5)	518/935 (55.4)
Male	753/1656 (45.5)	417/935 (44.6)
Education		
Less than high school	58/1656 (3.5)	68/935 (7.3)
High school	436/1656 (26.3)	317/935 (33.9)
Some college	507/1656 (30.6)	271/935 (29.0)
College degree or higher	655/1656 (39.6)	279/935 (29.8)
*APOE *ε4 allele status	375/1630 (23.0)	216/935 (23.1)
Current smokers	228/1656 (13.8)	87/935 (9.3)
Hypertension	591/1651 (35.8)	496/929 (53.4)
Diabetes	157/1644 (9.6)	139/926 (15.0)
Prevalent cardiovascular disease	139/1656 (8.4)	157/935 (16.8)
FSRP at examination 6, median (IQR)^a^	1.43 (0.66-4.08)	4.59 (2.56-8.13)
SBP at examination 6, mean (SD), mm Hg	126.0 (18.3)	134.3 (19.1)
PTA category		
Normal (0 to <16 dB HL)	926/1647 (56.2)	283/931 (30.4)
Slight (16 to <26 dB HL)	384/1647 (23.3)	289/931 (31.0)
Mild (26 to <40 dB HL)	252/1647 (15.3)	263/931 (28.3)
At least moderate (≥40 dB) hearing loss	87/1647 (5.3)	96/931 (10.3)
PTA, median (IQR), dB		
Overall	13.75 (8.75-23.75)	21.25 (13.75-31.25)
Men	11.25 (7.50-18.75)	17.50 (12.50-27.50)
Women	17.50 (11.25-27.50)	26.25 (18.75-33.75)
Neuropsychological measures		
PC1-6V score, mean (SD)	0.03 (0.97)	NA
Logical memory delay score, mean (SD)^b^	10.58 (3.60)	NA
Visual reproduction delay score, mean (SD)^c^	8.17 (3.37)	NA
Similarities score, mean (SD)^d^	16.84 (3.46)	NA
TMT-B − TMT-A, median (IQR), s	0.67 (0.45-0.98)	NA
Hopper Visual Organization Test score, median (IQR)^e^	25.5 (24-27)	NA
Time between auditory and cognitive examination, mean (SD), y	3.8 (1.1)	NA
MRI measures (n = 1508)		
Total intracranial volume, mean (SD), cm^3^	1253.1 (124.8)	NA
Total brain volume, mean (SD), cm^3^	971.7 (103.7)	NA
Total hippocampal volume, mean (SD), cm^3^	6.6 (0.7)	NA
White matter hyperintensities, median (IQR), cm^3^	0.6 (0.3-1.1)	NA
Time between and auditory examination and brain MRI measures, mean (SD), y	3.8 (1.1)	NA
Incident Alzheimer disease during 15-y follow-up	NA	91/935 (9.7)
Incident dementia during 15-y follow-up	NA	118/935 (12.6)

Abbreviations: *APOE*, apolipoprotein E; dB HL, decibels hearing loss; FHS, Framingham Heart Study; FSRP, Framingham Stroke Risk Profile; MRI, magnetic resonance imaging; NA, not applicable; PC1-6v, first principal component based on 6 variables; PTA, pure tone average; SBP, systolic blood pressure; TMT-A, Trail Making Test part A; TMT-B, Trail Making Test part B.

^a^ Scale of 0 to 30 or higher, with higher scores indicating greater 10-year stroke risk.

^b^ Scale of 0 to 25, with higher scores indicating better verbal memory (delayed recall).

^c^ Scale of 0 to 41, with higher scores indicating better visual memory.

^d^ Scale of 0 to 28 (raw), with higher scores indicating better abstract reasoning.

^e^ Scale of 0 to 30, with higher scores indicating better visuospatial integration.

Men had greater mean hearing loss than women (sample 1: median, 11.25 dB [IQR, 7.50-18.75 dB] vs 17.50 dB [IQR, 11.25-27.50 dB]; sample 2: median, 17.50 dB [IQR, 12.50-27.50 dB] vs 26.25 dB [IQR, 18.75-33.75 dB]; *P* < .001) (Table 1). The percentage of individuals with normal hearing decreased with age from 56.2% in sample 1 to 30.4% in sample 2. We observed substantial discrepancies between self-identified and objectively measured hearing loss showing the value of PTA for detecting mild hearing loss, although congruence improved with increasing severity of hearing loss such that most participants (78 of 85 [91.7%]) with moderate or greater hearing loss were aware of their deficit (eTable 1 in Supplement 1). A comparison among participants who attended examination 6 based on whether they were included in sample 1 is also presented in eTable 1 in Supplement 1. As expected, participants included in our study were, on average, younger and healthier.

Table 2 shows the associations between PTA thresholds and various MRI and neuropsychological measures both at baseline and over time. We found that increasing PTA thresholds were linearly associated with a greater increase in WMHV (β [SE], 0.02 [0.01]; *P* = .048) and a greater decline in TMT-B minus TMT-A performance (β [SE], −0.02 [0.01]; *P* = .02). Comparing participants with at least slight hearing loss with those with normal hearing, we observed a larger increase in WMHV (β [SE], 0.03 [0.01]; *P* = .03). Participants with at least mild hearing loss had a smaller TCBV at baseline than those with slight or no hearing loss (β [SE], –4.10 [1.76]; *P* = .02) and a greater decline in executive function (TMT-B minus TMT-A: β [SE], −0.04 [0.01]; *P* = .009). Additional adjustment for vascular risk factors (SBP, T2D, and current smoking status) did not significantly alter our results, although the slightly smaller sample size of participants with these covariates available accounted for slightly larger *P* values (eTable 2 in Supplement 1). Associations of objective hearing loss using an alternate threshold (participants with at least moderate hearing loss compared with all others) and of self-reported hearing loss with the same MRI measures as in Table 2 are shown in eTable 3 in Supplement 1. Self-reported hearing loss was associated with increasing WMHV (β [SE], 0.03 [0.01]; *P* = .03) and worsening executive performance (β [SE], −0.02 [0.01]; *P* = .04). Results of analyses examining PTA thresholds of other neuropsychological and MRI outcomes that were not significant are shown in eTables 4 to 7 in Supplement 1. In exploratory, hypothesis-generating analyses, we found interactions with sex and *APOE *ε4 genotype on the association between hearing loss and MRI and neuropsychological variables (eTable 8 in Supplement 1). At least slight hearing loss was associated with lower TCBV among men alone (β [SE], −5.26 [2.32]; *P* = .02) and with greater WMHV progression among women alone (β [SE], 0.06 [0.03]; *P* = .02), suggesting potential sex-specific vulnerabilities.

**Table 2.** Associations Between Best Ear PTA Thresholds^a^ and Hearing Loss Categories

Variable	TCBV on MRI at examination 7 (n = 1464)^b^		Annualized change in WMHV from examinations 7 to 8 (n = 1039)^c^		Annualized change in TMT-B − TMT-A from examinations 7 to 8 (n = 1218)^d^	
	β (SE)	*P* value	β (SE)	*P* value	β (SE)	*P* value
Continuous: log (best ear PTA)	−0.83 (1.21)	.49	0.02 (0.01)	.048	−0.02 (0.01)	.02
NHATS hearing loss categories, adjusted mean (SE)						
Normal (0 to <16 dB HL)	973.01 (0.90)	.07	0.13 (0.01)	.050	−0.05 (0.01)	.07
Slight (16 to <26 dB HL)	971.26 (1.32)		0.16 (0.01)		−0.05 (0.01)	
Mild (26 to <40 dB HL)	967.57 (1.73)		0.16 (0.02)		−0.08 (0.02)	
At least moderate (≥40 dB HL)	969.67 (0.90)		0.21 (0.03)		−0.09 (0.03)	
Comparison between NHATS categories						
At least slight hearing loss vs none (reference)	−2.94 (1.48)	.047	0.03 (0.01)	.03	−0.02 (0.01)	.18
At least mild vs at least slight hearing loss (reference)	−4.10 (1.76)	.02	0.03 (0.02)	.08	−0.04 (0.01)	.009

Abbreviations: dB HL, decibels hearing loss; MRI, magnetic resonance imaging; NHATS, National Health and Aging Trends Study; PTA, pure tone average; TCBV, total cerebral brain volume; TMT-A, Trail Making Test part A; TMT-B, Trail Making Test part B; WMHV, white matter hyperintensity volume.

^a^ Pure tone average thresholds were 500, 1000, 2000, and 4000 Hz.

^b^ Adjusted for age at MRI at examination 7, age squared, sex, time between audiometry at examination 6 and MRI at examination 7, and head size.

^c^ Natural log transformed, adjusted for age at MRI at examination 7, age squared, sex, time between audiometry at examination 6 and MRI at examination 7, and total brain volume.

^d^ Adjusted for age at neuropsychological testing at examination 7, age squared, sex, education, and time between audiometry at examination 6 and neuropsychological testing at examination 7.

### Association Between Objective Hearing Loss at Examination 6 and Incident Dementia

**Figure 2.**
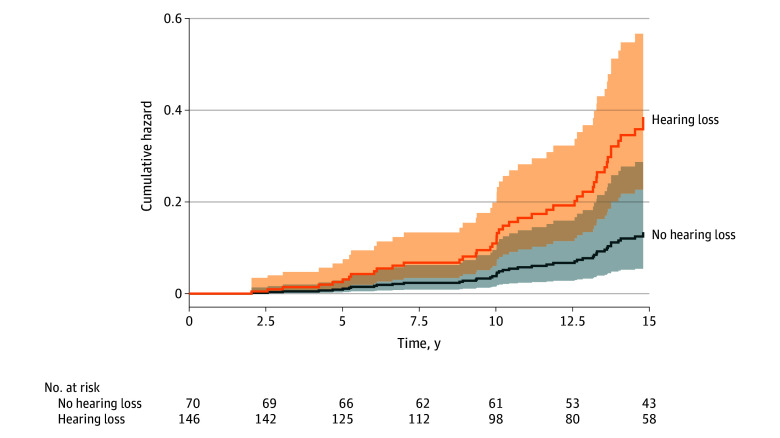
Cumulative Hazard Curves Comparing Apolipoprotein ε4 (*APOE4*) Carriers With and Without Hearing Loss Model adjusted for age, sex, and education. Shaded areas indicate 95% CIs.

During the 15-year follow-up period, 118 of 931 participants in sample 2 (12.7%) were diagnosed with incident dementia, of whom 91 (77.1%) had Alzheimer disease. We observed a higher risk of developing any dementia in participants with at least slight hearing loss compared with those with normal hearing (HR, 1.71; 95% CI, 1.01-2.90; *P* = .045) (Table 3) and observed a continuous association between higher PTA thresholds in the better ear and greater dementia risk (HR, 2.23; 95% CI, 1.13-4.40; *P* = .02). There was a significant interaction between hearing loss and *APOE* ε4 carrier status such that participants with at least slight hearing loss had a greater risk of dementia compared with participants with normal hearing (HR, 2.86; 95% CI, 1.12-7.28; *P* = .03) (Table 3; Figure 2). We also observed that self-reported hearing loss was associated with an increased risk of dementia among participants with an *APOE* ε4 allele (HR, 1.94; 95% CI, 1.03-3.65; *P* = .04) (eTable 9 in Supplement 1); this association between hearing loss and dementia risk persisted in *APOE* ε4 carriers after additional adjustment for vascular risk factors SBP, T2D, and current smoking status (HR, 2.61; 95% CI, 1.02-6.70; *P* = .046) (eTable 10 in Supplement 1). Furthermore, adjustment for the competing risk of death did not change our primary results (eTable 11 in Supplement 1).

**Table 3.** Associations Between Best Ear PTA Thresholds^a^ and Hearing Loss Categories at Examination 6 and 15-Year Follow-Up From Examination 7 to Incident Dementia

Variable	Incident dementia, full sample^b^		Incident dementia, stratified by *APOE *ε4 status^c^				Incident Alzheimer disease^b^	
			Negative		Positive			
	HR (95% CI)	*P* value	HR (95% CI)	*P* value	HR (95% CI)	*P* value	HR (95% CI)	*P* value
No. of participants/total No.	118/931	NA	73/715	NA	45/216	NA	91/931	NA
Continuous: log (PTA in best ear)	1.23 (0.83-1.84)	.31	0.92 (0.57-1.50)	.74	2.23 (1.13-4.40)	.020	1.31 (0.83-2.06)	.25
NHATS categories								
Normal (0 to <16 dB HL)	1 [Reference]	.17	1 [Reference]	.26	1 [Reference]	.12	1 [Reference]	.41
Slight (16 to <26 dB HL)	1.67 (0.95-2.93)		1.35 (0.67-2.73)		2.83 (1.06-7.56)		1.42 (0.75-2.69)	
Mild (26 to <40 dB HL)	1.86 (1.04-3.33)		1.60 (0.80-3.22)		2.61 (0.91-7.19)		1.75 (0.92-3.36)	
At least moderate (≥40 dB HL)	1.40 (0.66-2.98)		0.78 (0.29-2.14)		4.13 (1.21-14.04)		149 (0.65-3.43)	
Overall comparison of NHATS categories								
Slight hearing loss or worse vs no hearing loss	1.71 (1.01-2.90)	.045	1.39 (0.74-2.63)	.31	2.86 (1.12-7.28)	.03	1.55 (0.87-2.78)	.14

Abbreviations: *APOE*, apolipoprotein E; dB HL, decibels hearing loss; HR, hazard ratio; NA, not applicable; NHATS, National Health and Aging Trends Study; PTA, pure tone average.

^a^ Pure tone average thresholds were 500, 1000, 2000, and 4000 Hz.

^b^ Adjusted for age at examination 6, sex, education, and *APOE *ε4.

^c^ Adjusted for age at examination 6, sex, and education.

### Hearing Aid Use Association With Hearing Loss and Incident Dementia

Among participants with at least slight hearing loss, those who did not use hearing aids had an elevated risk of dementia (HR, 1.72; 95% CI, 1.02-2.91; *P* = .04), whereas those who used hearing aids had a smaller, nonsignificant increased risk. Again, the association was primarily seen in *APOE* ε4 carriers (HR, 2.82; 95% CI, 1.11-7.16; *P* = .03) (eTable 12 in Supplement 1).

### Inclusion of Hearing Loss in Estimating Dementia Risk

We examined whether we could improve on a standard dementia risk prediction model by including hearing loss as a factor, alongside age, sex, education, and *APOE4* genotype. The addition of hearing loss was associated with a modest increase in the integrated discrimination index (IDI) of 0.007 (95% CI, 0.003-0.017) and a net reclassification index (NRI) of 0.235 (95% CI, 0.059-0.419). The incremental value of adding hearing loss data remained substantive when the base model included vascular risk factors of SBP, T2D, and current smoking status, with an IDI of 0.006 (95% CI, 0.002-0.010) and NRI of 0.24 (95% CI, 0.061-0.420).

## Discussion

This cohort study found that objective hearing loss of more than a slight degree was associated with a 70% or greater increase in the risk of dementia, in accordance with prior studies that also considered hearing loss as a risk factor for developing dementia.^1,18,19,47,48^ We also found that hearing loss was associated with accelerated aging in the form of lower brain volume and with vascular brain aging as indicated by an increase in WMHV and a vascular pattern of cognitive impairment involving a decline in executive function. Identifying the earliest neurologic correlates of hearing loss may help with understanding the biological pathways involved.

We explored whether there were modifying factors for these associations. Associations were observed on brain MRI in women and with declines in cognitive performance among participants aged 60 years or older. The risk of dementia among participants with hearing loss was much greater in *APOE* ε4 allele carriers than in those without an *APOE* ε4 allele, an interesting gene-environment interaction. Our findings showed that age, sex, and genetics may influence the association between hearing loss and brain health.

We compared self-identified hearing loss and threshold sensitivity losses revealed through audiometric testing and observed excellent concordance between the 2 among participants with at least moderate hearing loss in the better ear. However, among participants with mild or lesser degrees of hearing loss, many appeared unaware of their hearing loss. Some participants who self-reported hearing loss in the absence of objectively impaired PTA may have been experiencing declines in hearing functions, such as speech discrimination, that are imperfectly captured by threshold metrics.^49,50,51^ These results suggest a role for audiometric testing to complement self-reports since hearing loss of less than moderate severity was also associated with adverse changes on brain MRI, poorer cognition, and increased dementia risk, and hearing aid use may, as our data suggest, mitigate these adverse effects.^52,53^

We evaluated whether including objective hearing loss as a risk factor improved the estimative accuracy of a dementia prediction model based on age, sex, and *APOE *ε4 genotype and found that it did. Increases in both the IDI and NRI suggested modest improvement in classification accuracy in distinguishing between people who may or may not develop dementia, comparable to the improvement in estimative accuracy noted when adding plasma levels of amyloid β42 to a baseline dementia prediction algorithm^54^ or of adding plasma vascular endothelial growth factor (or C-reactive protein and homocysteine combined) to a baseline 10-year stroke risk prediction model.^55^ This improvement was attenuated but still observed after additional inclusion of various vascular risk factors (SBP, T2D, and current smoking status) in the base model. In our study, the observed improvements in IDI and NRI were greater in magnitude to the improvement Chouraki et al^56^ observed by incorporating an early version of a polygenic Alzheimer disease genetic risk score into an age- and sex-based model predicting incident Alzheimer disease (NRI, 0.121; IDI, 0.009).

There are several possible explanations for the associations we observed. It is possible that hearing loss is associated with loneliness and social isolation,^57,58,59,60^ subtle changes in brain connectivity with the decrement in auditory stimulation, or a greater demand on the brain because of the need for more attention during auditory decoding.^60,61,62,63,64^ An alternative explanation may be an ascertainment bias since persons with poorer hearing might perform worse on tests of cognition and, therefore, be more likely to be diagnosed with dementia.^65,66,67,68^ The association we observed with brain MRI measures, however, cannot be attributed to any such ascertainment bias.

Hearing loss becomes more common as people age.^69,70^ From a public health perspective, hearing loss has been identified as a major modifiable risk factor for dementia, perhaps accounting for 8% of the 10 million new cases of dementia diagnosed each year.^7^ In terms of public health impact, if the association is shown to be causal, managing hearing loss may reduce the risk of cognitive decline and dementia. If the association is explained by vascular factors impacting both hearing and risk of dementia, more aggressive management of vascular risk factors as soon as hearing loss is detected might reduce both further hearing loss and risk of dementia. The intriguing observations of an association of hearing loss with dementia in *APOE* ε4 carriers suggest that targeting assessment and correction of hearing loss to this at-risk subgroup may be particularly cost-effective.

### Limitations

This study had several limitations. Since it was an observational study, we cannot infer causality; however, hearing loss was diagnosed in participants with no cognitive impairments and examined relative to subsequent incident dementia. The observation of an association of hearing loss with dementia in participants carrying the *APOE* ε4 allele needs replication due to the small subsample size. That there was an association between hearing loss and structural changes on brain MRI, not just cognitive changes, suggests that the observed associations are not merely reflecting worse performance because of difficulty understanding the instructions for testing.^71^ Thus, we believe that our findings may not be purely because of ascertainment bias and that further investigation might reveal causation.^72^ It is possible that vascular risk factors may be responsible for both hearing loss and dementia, but adjusting for several major vascular risk factors did not alter our findings.^60^ The observation that hearing aid use was associated with a lower risk of dementia in participants with hearing loss also suggested both direct and indirect influences. Our observation that a simple test of hearing improved estimates of dementia risk may be valuable in a geriatric setting.^16,19^ Even if improving hearing does not reduce the risk of dementia in older adults, more accurate risk prediction tools may help clinicians advise their patients.^19,47^ We acknowledge that some of the observed associations may not survive correction for multiple testing and should be considered exploratory to be replicated in other cohorts.

## Conclusions

This cohort study highlights a significant association between hearing loss and smaller brain volume, with an accelerated accrual of white matter abnormalities, an accelerated decline in executive function, and a near tripling of dementia risk among persons carrying an *APOE* ε4 allele that is partially mitigated by hearing aid use. The findings suggest that testing for hearing loss may be helpful in identifying potential future risk of dementia. The association patterns between hearing loss and accelerated accrual of white matter hyperintensities point toward vascular brain injury as a possible pathway between hearing loss and cognitive decline, but the association persisted after adjustment for major vascular risk factors, and other potential pathways remain to be explored. Overall, this study underscores the importance of addressing hearing loss not only as a quality-of-life issue but also as an important factor in maintaining brain health and reducing the risk of dementia.

## Notes

Supplement 1. **eTable 1.** Comparison Between Self-Identified Hearing Loss and Hearing Loss by PTA at Examination 6 **eTable 2.** Association of Best Ear PTA Thresholds as Continuous Variables and Hearing Loss Categories at Examination 6, With MRI Outcomes at Examination 7 and Annualized Change in MRI and NP From Examinations 7 and 8, Additionally Adjusted for Smoking, Systolic Blood Pressure, and Diabetes **eTable 3. **Associations of Best Ear Pure Tone Average (PTA) Thresholds (500, 1000, 2000, and 4000 Hz) Measuring Hearing Loss (HL) of Moderate or Greater Severity vs Mild or Lesser HL and of Self-Reported HL, All at Examination 6 With MRI Outcomes at Examination 7 and Annualized Change in MRI and NP From Examinations 7 to 8 **eTable 4.** Nonsignificant Associations Between Best Ear Pure Tone Average (PTA) Thresholds (500, 1000, 2000, and 4000 Hz) and Hearing Loss (HL) at Examination 6 and Baseline NP Outcomes at Examination 7 **eTable 5.** Associations Between Best Ear Pure Tone Average (PTA) Thresholds (500, 1000, 2000, and 4000 Hz) and Hearing Loss (HL) at Examination 6 and Annualized Change in NP Outcomes From Examination 7 to Examination 8 **eTable 6.** Nonsignificant Associations Between Best Ear Pure Tone Average (PTA) Thresholds (500, 1000, 2000, and 4000 Hz) and Hearing Loss (HL) at Examination 6 and MRI Outcome at Baseline **eTable 7.** Associations Between Best Ear Pure Tone Average (PTA) Thresholds (500, 1000, 2000, and 4000 Hz) and Hearing Loss (HL) at Examination 6 and Annualized Change in MRI Outcomes From Examination 7 to Examination 8 **eTable 8.** Stratified Results for Analyses With Significant Interaction in Relating Hearing (PTA) to MRI and NP Measures **eTable 9.** Association Between Best Ear Pure Tone Average (PTA) Thresholds (500, 1000, 2000, and 4000 Hz), Measured Hearing Loss (HL), and Self-Reported HL at Examination 6 and 15-Year Follow-Up From Examination 7 to Incident Dementia **eTable 10.** Association Between Best Ear Pure Tone Average (PTA) Thresholds (500, 1000, 2000, and 4000 Hz) and Hearing Loss (HL) Categories at Examination 6 and 15-Year Follow-Up From Examination 7 to Incident Dementia, Additionally Adjusted for Systolic Blood Pressure, Diabetes, and Smoking **eTable 11.** Association Between Best Ear Pure Tone Average (PTA) Thresholds (500, 1000, 2000, and 4000 Hz) and Hearing Loss (HL) Categories at Examination 6 and 15-Year Follow-Up From Examination 7 to Incident Dementia, Adjusted for the Competing Risk of Death **eTable 12.** Association Between Hearing Loss Defined as At Least Slight HL (PTA ≥16 dB), Hearing Aid Use, and 15-Year Follow-Up From Examination 7 to Incident Dementia

Supplement 2.
Data Sharing Statement
