# Supplementary material for: Hearing Loss, Brain Structure, Cognition, and Dementia Risk in the Framingham Heart Study
Source: JAMA Netw Open. 2025 Nov 5;8(11):e2539209. doi: 10.1001/jamanetworkopen.2025.39209 (PMC12590305; doi:10.1001/jamanetworkopen.2025.39209)
Supplement: Supplement 1. — eTable 1. Comparison Between Self-Identified Hearing Loss and Hearing Loss by PTA at Examination 6 eTable 2. Association of Best Ear PTA Thresholds as Continuous Variables and Hearing Loss Categories at Examination 6, With MRI Outcomes at Examination 7 and Annualized Change in MRI and NP From Examinations 7 and 8, Additionally Adjusted for Smoking, Systolic Blood Pressure, and Diabetes eTable 3. Associations of Best Ear Pure Tone Average (PTA) Thresholds (500, 1000, 2000, and 4000 Hz) Measuring Hearing Loss (HL) of Moderate or Greater Severity vs Mild or Lesser HL and of Self-Reported HL, All at Examination 6 With MRI Outcomes at Examination 7 and Annualized Change in MRI and NP From Examinations 7 to 8 eTable 4. Nonsignificant Associations Between Best Ear Pure Tone Average (PTA) Thresholds (500, 1000, 2000, and 4000 Hz) and Hearing Loss (HL) at Examination 6 and Baseline NP Outcomes at Examination 7 eTable 5. Associations Between Best Ear Pure Tone Average (PTA) Thresholds (500, 1000, 2000, and 4000 Hz) and Hearing Loss (HL) at Examination 6 and Annualized Change in NP Outcomes From Examination 7 to Examination 8 eTable 6. Nonsignificant Associations Between Best Ear Pure Tone Average (PTA) Thresholds (500, 1000, 2000, and 4000 Hz) and Hearing Loss (HL) at Examination 6 and MRI Outcome at Baseline eTable 7. Associations Between Best Ear Pure Tone Average (PTA) Thresholds (500, 1000, 2000, and 4000 Hz) and Hearing Loss (HL) at Examination 6 and Annualized Change in MRI Outcomes From Examination 7 to Examination 8 eTable 8. Stratified Results for Analyses With Significant Interaction in Relating Hearing (PTA) to MRI and NP Measures eTable 9. Association Between Best Ear Pure Tone Average (PTA) Thresholds (500, 1000, 2000, and 4000 Hz), Measured Hearing Loss (HL), and Self-Reported HL at Examination 6 and 15-Year Follow-Up From Examination 7 to Incident Dementia eTable 10. Association Between Best Ear Pure Tone Average (PTA) Thresholds (500, 1000, 2000, and 4000 Hz) [file jamanetwopen-e2539209-s001.pdf]

Supplemental Online Content

Kolo FB, Lu S, Beiser AS, et al. Hearing loss, brain structure, cognition, and dementia risk in the Framingham Heart Study. *JAMA Netw Open*. 2025;8(10):e2539209.
doi:10.1001/jamanetworkopen.2025.39209

eTable 1. Comparison Between Self-Identified Hearing Loss and Hearing Loss by PTA at Examination 6

eTable 2. Association of Best Ear PTA Thresholds as Continuous Variables and Hearing Loss Categories at Examination 6, With MRI Outcomes at Examination 7 and Annualized Change in MRI and NP From Examinations 7 and 8, Additionally Adjusted for Smoking, Systolic Blood Pressure, and Diabetes

eTable 3. Associations of Best Ear Pure Tone Average (PTA) Thresholds (500, 1000, 2000, and 4000 Hz) Measuring Hearing Loss (HL) of Moderate or Greater Severity vs Mild or Lesser HL and of Self-Reported HL, All at Examination 6 With MRI Outcomes at Examination 7 and Annualized Change in MRI and NP From Examinations 7 to 8

eTable 4. Nonsignificant Associations Between Best Ear Pure Tone Average (PTA) Thresholds (500, 1000, 2000, and 4000 Hz) and Hearing Loss (HL) at Examination 6 and Baseline NP Outcomes at Examination 7

eTable 5. Nonsignificant Associations Between Best Ear Pure Tone Average (PTA) Thresholds (500, 1000, 2000, and 4000 Hz) and Hearing Loss (HL) at Examination 6 and Annualized Change in NP Outcomes From Examination 7 to Examination 8

eTable 6. Nonsignificant Associations Between Best Ear Pure Tone Average (PTA) Thresholds (500, 1000, 2000, and 4000 Hz) and Hearing Loss (HL) at Examination 6 and MRI Outcome at Baseline

eTable 7. Associations Between Best Ear Pure Tone Average (PTA) Thresholds (500, 1000, 2000, and 4000 Hz) and Hearing Loss (HL) at Examination 6 and Annualized Change in MRI Outcomes From Examination 7 to Examination 8

eTable 8. Stratified Results for Analyses With Significant Interaction in Relating Hearing (PTA) to MRI and NP Measures

eTable 9. Association Between Best Ear Pure Tone Average (PTA) Thresholds (500, 1000, 2000, and 4000 Hz), Measured Hearing Loss (HL), and Self-Reported HL at Examination 6 and 15-Year Follow-Up From Examination 7 to Incident Dementia

eTable 10. Association Between Best Ear Pure Tone Average (PTA) Thresholds (500, 1000, 2000, and 4000 Hz) and Hearing Loss (HL) Categories at Examination 6 and 15-Year Follow-Up From Examination 7 to Incident Dementia, Additionally Adjusted for Systolic Blood Pressure, Diabetes, and Smoking

eTable 11. Association Between Best Ear Pure Tone Average (PTA) Thresholds (500, 1000, 2000, and 4000 Hz) and Hearing Loss (HL) Categories at Examination 6 and 15-Year Follow-Up From Examination 7 to Incident Dementia, Adjusted for the Competing Risk of Death

eTable 12. Association Between Hearing Loss Defined as At Least Slight HL (PTA ≥16 dB), Hearing Aid Use, and 15-Year Follow-Up From Examination 7 to Incident Dementia

This supplemental material has been provided by the authors to give readers additional information about their work.

© 2025 Kolo FB et al. *JAMA Network Open*.

eTable 1. Comparison Between Self-Identified Hearing Loss and Hearing Loss by PTA at Examination 6

**Table 1a. Comparison between Self-identified Hearing Loss and Any Objective Hearing Loss (PTA ≥16 dB) in Framingham Offspring at Examination 6**			
**Objective Hearing Loss: Any (At Least Slight) Hearing Loss**	**Self-identified Hearing Loss**		
	Yes	No	Total
Yes	377 (53%)	341	718
No	102 (11%)	817	919

**Table 1b. Comparison between Self-identified Hearing Loss and At Least Mild Objective Hearing Loss (≥26 dB) in Framingham Offspring at Examination 6**			
**Objective Hearing Loss: At Least Mild Hearing Loss**	**Self-identified Hearing Loss**		
	Yes	No	Total
Yes	242 (72%)	94	336
No	237 (18%)	1064	1301

**Table 1c. Comparison between Self-identified hearing loss and At Least Moderate Objective Hearing Loss (>40 dB) in Framingham Offspring at Examination 6**			
**Objective Hearing Loss: At Least Moderate Hearing Loss**	**Self-identified Hearing Loss**		
	Yes	No	Total
Yes	78 (92%)	7	85
No	401 (26%)	1151	1552

© 2025 Kolo FB et al. *JAMA Network Open*.

Table 1d. Baseline characteristics of the Framingham Heart Study Offspring cohort participants who attended Examination 6 according to whether or not they were included in the cognitive assessment sample (Sample 1).

**Characteristics**	**Study sample 1 (n=1656)**	**Examination Attendees Not in Study Sample (persons without Hearing/ NP information) (n=1876)**
Age, years, mean (range)**	58.06 [29.67, 85.55]	60.47 [33.09, 86.74]
Female, n/total (%)	903 (54.53)	972/1876 (51.81)
**Education, n/total, (%)**		
< High school degree	58/1656 (3.50)	147/1807 (8.14)
High school degree	436/1656 (26.33)	629/1807 (34.81)
Some college	507/1656 (30.62)	496/1807 (27.45)
College degree or higher	655/1656 (39.55)	535/1807 (29.61
APOE-4 allele status, n/total (%)	375/1618 (23.01)	376/1717 (21.78)
Current Smoking, n/total (%)**	228/1656 (13.77)	312/ 1876 (16.63)
Systolic Blood Pressure, mean (sd)**	125.98 (18.27)	130.71 (18.89)
Diabetes, n/total (%)**	157/1644 (9.55)	233/1788 (13.03)
Prevalent cardiovascular disease, n/total (%)**	139/1656 (8.39)	274/1876 (14.61)
FSRP at Examination 6, median [IQR]**	1.43 [0.66, 4.08]	2.31 [0.88, 6.24]

*-p-values<0.05, **-p-values<0.01; the FSRP (Framingham Stroke Risk Profile) is a score that describes the 10-year risk of developing an incident stroke as a percentage.

eTable 2. Association of Best Ear PTA Thresholds as Continuous Variables and Hearing Loss Categories at Examination 6, With MRI Outcomes at Examination 7 and Annualized Change in MRI and NP From Examinations 7 and 8, Additionally Adjusted for Smoking, Systolic Blood Pressure, and Diabetes

	**Total cerebral brain volume (TCB)* at examination 7 (n=1454)**		**Annualized Change in White matter hyperintensity volume (WMH)** from examination 7 to 8 (n=1036)**		**Annualized Change in Trails B-Trails A (TrB-TrA)*** From examination 7 to 8 (n=1215)**	
	**β±SE**	p-value	**β±SE**	p-value	**β±SE**	p-value
**Continuous: log(best ear PTA)**	-0.32±1.22	0.79	0.02±0.01	0.06	**-0.02±0.01**	**0.024**
**≥Slight HL vs. None**	-2.55±1.48	0.09	**0.03±0.01**	**0.037**	-0.02±0.01	0.16
**≥Mild HL vs. ≤Slight HL (ref)**	**-3.89±1.77**	**0.028**	0.03±0.02	0.06	**-0.04±0.01**	**0.012**

*Adjusted for Age at MRI at Examination 7, Age^2, Sex, Time between audiometry at exam 6 and MRI at Examination 7, and cerebrum cranial volume
**Natural log-transformed, adjusted for Age at MRI at Examination 7, Age^2, Sex, Time between audiometry at exam 6 and MRI at Examination 7, and total brain volume as a proxy measure of head size
*** Adjusted for Age at NP test at Examination 7, Age^2, Sex, Education, and Time between audiometry at Examination 6 and NP at Examination 7.

© 2025 Kolo FB et al. *JAMA Network Open*.

eTable 3. Associations of Best Ear Pure Tone Average (PTA) Thresholds (500, 1000, 2000, and 4000 Hz) Measuring Hearing Loss (HL) of Moderate or Greater Severity vs Mild or Lesser HL and of Self-Reported HL, All at Examination 6 With MRI Outcomes at Examination 7 and Annualized Change in MRI* and NP From Examinations 7 to 8 , same outcomes as shown in Table 2**

	**Total cerebral brain volume (TCB) at examination 7 (n=1452)**		**Annualized Change in White matter hyperintensity volume (WMH) from examination 7 to 8 (n=1033)**		**Annualized Change in Trails B-Trails A (TrB-TrA) From examination 7 to 8 (n=1211)**	
	**β±SE**	p-value	**β±SE**	p-value	**β±SE**	p-value
**≥Moderate HL vs. ≤Mild HL (ref)**	-1.12±2.92	0.70	**0.06±0.03**	**0.046**	-0.03±0.03	0.19
**Self-reported HL vs none**	-1.84±1.43	0.20	**0.03±0.01**	**0.033**	**-0.02±0.01**	**0.042**

*Adjusted for Age at MRI at examination 7, Age^2, Sex, Time between audiometry at examination 6 and MRI at examination 7, and headsize
**Natural log-transformed, adjusted for Age at MRI at examination 7, Age^2, Sex, Time between audiometry at examination 6 and MRI at examination 7, and total brain
*** Adjusted for Age at NP test at examination 7, Age^2, Sex, Education, and Time between audiometry at examination 6 and NP at examination 7.
The number of persons studied is slightly lower than in Table 2 since only persons with self-reported HL data available are included in this analysis

© 2025 Kolo FB et al. *JAMA Network Open*.

eTable 4. Nonsignificant Associations Between Best Ear Pure Tone Average (PTA) Thresholds (500, 1000, 2000, and 4000 Hz) and Hearing Loss (HL) at Examination 6 and Baseline NP Outcomes at Examination 7*						
	Trails B-Trails A** (n=1632)		Logical Memories – Delayed (LMd) (n=1648)		General Cognitive Performance PC1-6V (n=1624)	
	**Beta±SE**	**p-value**	**Beta±SE**	**p-value**	**Beta±SE**	**p-value**
Continuous: log (best ear PTA)	-0.01±0.01	0.34	-0.06±0.16	0.73	-0.02±0.04	0.56
≥Slight HL vs. None	-0.02±0.01	0.14	-0.25±0.20	0.21	-0.05±0.05	0.28
≥Mild HL vs. ≤Slight HL (ref), β±SE	-0.01±0.01	0.26	0.13±0.23	0.58	0.01±0.05	0.90
*Adjusted for Age at NP test at Examination 7, Age^2, Sex, Education and Time between NP at examination 7.<br>**log transformed						

eTable 5. Nonsignificant Associations Between Best Ear Pure Tone Average (PTA) Thresholds (500, 1000, 2000, and 4000 Hz) and Hearing Loss (HL) at Examination 6 and Annualized Change in NP Outcomes* From Examination 7 to Examination 8				
**Best Ear PTA Thresholds**	**Logical Memory – Delayed (LMd) (n=1269)**		**General Cognitive Performance PC1-6V (n=1163)**	
	**Beta±SE**	**p-value**	**Beta±SE**	**p-value**
Continuous: log (best ear PTA)	0.03±0.03	0.40	-0.01±0.01	0.32
≥Slight HL vs. None	-0.05±0.04	0.25	0.00±0.01	0.81
≥Mild HL vs. ≤Slight HL (ref**), β±SE**	-0.05±0.05	0.31	-0.02±0.01	0.07
* Adjusted for Age at NP test at Examination 7, Age^2, Sex, Education and Time between NP at examination.				

© 2025 Kolo FB et al. *JAMA Network Open*.

eTable 6. Nonsignificant Associations Between Best Ear Pure Tone Average (PTA) Thresholds (500, 1000, 2000, and 4000 Hz) and Hearing Loss (HL) at Examination 6 and MRI Outcome at Baseline				
	White matter hyperintensity volume (WMHV)** (n=1446)		Hippocampal Volume (n=1464)	
	Beta±SE	p-value	Beta±SE	p-value
Continuous: log (best ear PTA)	-0.03±0.04	0.45	-0.002 ± 0.03	0.93
**≥Slight HL vs. None**	0.01±0.05	0.83	-0.05±0.03	0.16
≥Mild HL vs. ≤Slight HL (ref)	-0.01±0.06	0.83	-0.03±0.04	0.42

* Adjusted for Age at MRI at Examination 7, Age^2, Sex, Time between MRI at examination 7.
HPV additionally adjusted for intracranial volume; WMH additionally adjusted for total brain volume, both being proxy measures for head size

eTable 7. Associations Between Best Ear Pure Tone Average (PTA) Thresholds (500, 1000, 2000, and 4000 Hz) and Hearing Loss (HL) at Examination 6 and Annualized Change in MRI* Outcomes From Examination 7 to Examination 8				
	Total cerebrum brain volume (TCBV) (n=1051)		Hippocampal volume (HPV) (n=1051)	
	Beta±SE	p-value	Beta±SE	p-value
Continuous: log (best ear PTA)	-0.14±0.17	0.41	0.000±0.003	0.94
**≥Slight HL vs. None**	-0.34±0.21	0.11	-0.003±0.003	0.40
≥Mild HL vs. ≤Slight HL (ref)	-0.46±0.26	0.08	-0.000±0.004	0.95

*Adjusted for Age at MRI at Examination 7, Age^2, Sex, Time between MRI at examination 7.
TCB and HPV additionally adjusted for intracranial volume; WMH additionally adjusted for total brain volume, both being proxy measures of head size

© 2025 Kolo FB et al. *JAMA Network Open*.

eTable 8. Stratified Results for Analyses With Significant Interactions in Relating Hearing (PTA) to MRI and NP Measures					
**Association between Examination 6 PTA and Examination 7 TCB***					**Interaction p-value**
	Women (n=797)		Men (n=667)		
	β±SE	p-value	β±SE	p-value	
**≥Slight HL vs. None**	-0.42±1.84	0.82	**-5.26±2.32**	**0.024**	**0.099**
**Association between Examination 6 PTA and Hearing Loss and Annualized Change in WMHV from Examination 7 to Exam 8****					
	Women (n=569)		Men (n=470)		
	β±SE	p-value	β±SE	p-value	
**≥Mild HL vs. ≤Slight HL (ref)**	**0.06±0.03**	**0.020**	0.00±0.02	0.96	**0.08**

*Adjusted for Age at MRI at exam 7, Age^2, Sex, Time between audiometry at exam 6 and MRI measure at exam 7 of total cerebral brain volume

**Natural log-transformed, adjusted for Age at MRI at exam 7, Age^2, Sex, Time between audiometry at exam 6 and MRI at exam 7, and total brain

© 2025 Kolo FB et al. *JAMA Network Open*.

**eTable 9. Association Between Best Ear Pure Tone Average (PTA) Thresholds (500, 1000, 2000, and 4000 Hz), Measured Hearing Loss (HL), and Self-Reported HL at Examination 6 and 15-Year Follow-Up From Examination 7 to Incident Dementia**								
	**Incident Dementia, Full Sample ***		**Incident Dementia, Stratified by ApoE4 Status****				**Incident Alzheimer's Disease ***	
Cases/N	(117/920)		ApoE4 – (72/707)		ApoE4 + (45/213)		(90/920)	
	HR (95% CI)	p-value	HR (95% CI)	p-value	HR (95% CI)	p-value	HR (95% CI)	p-value
**≥Moderate HL vs. ≤Mild HL (ref)**	0.86 (0.48, 1.55)	0.62	**0.55 (0.24, 1.25)**	**0.15**	**1.82 (0.77, 4.33)**	**0.18**	1.02 (0.53, 1.93)	0.96
**Self-reported HL vs none**	0.89 (0.61, 1.31)	0.56	**0.56 (0.34, 0.94)**	**0.028**	**1.94 (1.03, 3.65)**	**0.040**	1.08 (0.70, 1.69)	0.73
* Adjusted for Age at Examination 6, Sex, Education, and APOE ε4<br>** Adjusted for Age at Examination 6, Sex, and Education								

**eTable 10. Association Between Best Ear Pure Tone Average (PTA) Thresholds (500, 1000, 2000, and 4000 Hz) and Hearing Loss (HL) Categories at Examination 6 and 15-Year Follow-Up From Examination 7 to Incident Dementia, Additionally Adjusted for Systolic Blood Pressure, Diabetes, and Smoking**								
	**Incident Dementia, Full Sample ***		**Incident Dementia, Stratified by APOE-4 Status****				**Incident Alzheimer's Disease ***	
Cases/N	**(118/922)**		APOE-4 - **(73/709)**		APOE-4 + **(45/213)**		**(91/922)**	
	HR (95% CI)	p-value	HR (95% CI)	p-value	HR (95% CI)	p-value	HR (95% CI)	p-value
**Continuous: log (PTA in best ear)**	1.21 (0.80, 1.81)	0.37	0.94 (0.57, 1.56)	0.80	**2.05 (1.03, 4.06)**	**0.041**	1.27 (0.80, 2.03)	0.31
**≥Slight HL vs. None**	1.61 (0.95, 2.72)	0.08	1.32 (0.70, 2.50)	0.39	**2.61 (1.02, 6.70)**	**0.046**	1.44 (0.80, 2.59)	0.22
* Adjusted for Age at Examination 6, Sex, Education, and APOE ε4<br>** Adjusted for Age at Examination 6, Sex, and Education								

© 2025 Kolo FB et al. *JAMA Network Open*.

**eTable 11. Association Between Best Ear Pure Tone Average (PTA) Thresholds (500, 1000, 2000, and 4000 Hz) and Hearing Loss (HL) Categories at Examination 6 and 15-Year Follow-Up From Examination 7 to Incident Dementia, Adjusted for the Competing Risk of Death***								
	**Incident Dementia, Full Sample ***		**Incident Dementia, Stratified by ApoE4 Status****				**Incident Alzheimer's Disease ***	
Cases/N	**(118/931)**		ApoE4 - (73)		ApoE4 + (45/216)		**(91/931)**	
	HR (95% CI)	p-value	HR (95% CI)	p-value	HR (95% CI)	p-value	HR (95% CI)	p-value
**Continuous: log (PTA in best ear)**	1.21 (0.83, 1.77)	0.33	0.93 (0.58,1.49)	0.75	**2.00 (1.13, 3.57)**	**0.018**	1.29 (0.82, 2.01)	0.27
≥Slight HL vs. None	**1.71 (1.01, 2.91)**	**0.047**	1.45 (0.78, 2.73)	0.24	**2.84 (1.08, 7.48)**	**0.035**	1.54 (0.85, 2.79)	0.16

* Adjusted for Age at Examination 6, Sex, Education, and APOE ε4
** Adjusted for Age at Examination 6, Sex, and Education
*****Using Fine-Gray subdistribution hazards**

© 2025 Kolo FB et al. *JAMA Network Open*.

eTable 12. Association Between Hearing Loss Defined as At Least Slight HL (PTA ≥16 dB), Hearing Aid Use, and 15-Year Follow-Up From Examination 7 to Incident Dementia

	**Incident Dementia, Full Sample ***		**Incident Dementia, Stratified by ApoE4 Status****				**Incident Alzheimer's Disease ***	
Cases/N	**(118/925)**		ApoE4 - (73/711)		ApoE4 + (45/214)		**(91/925)**	
	HR (95% CI)	p-value	HR (95% CI)	p-value	HR (95% CI)	p-value	HR (95% CI)	p-value
Normal Hearing and No Hearing Aid	1.00 (ref)		1.00 (ref)		1.00 (ref)		1.00 (ref)	
Hearing Loss and Hearing Aid	1.49 (0.70, 3.19)	0.30	1.22 (0.47, 3.13)	0.68	2.48 (0.67, 9.13)	0.17	1.33 (0.56, 3.12)	0.52
Hearing Loss and No Hearing Aid	**1.72 (1.02, 2.91)**	**0.043**	1.40 (0.74, 2.65)	0.30	**2.82 (1.11, 7.16)**	**0.030**	1.56 (0.87, 2.79)	0.14

* Adjusted for Age at Examination 6, Sex, Education, and APOE ε4

** Adjusted for Age at Examination 6, Sex, and Education

© 2025 Kolo FB et al. *JAMA Network Open*.
