# Supplementary material for: Hearing Loss, Brain Structure, Cognition, and Dementia Risk in the Framingham Heart Study
Source: JAMA Netw Open. 2025 Nov 5;8(11):e2539209. doi: 10.1001/jamanetworkopen.2025.39209 (PMC12590305; doi:10.1001/jamanetworkopen.2025.39209)
Supplement: Supplement 2. — Data Sharing Statement [file jamanetwopen-e2539209-s002.pdf]

Data Sharing Statement

Data
Data available: Yes
Data types: Deidentified participant data
How to access data: In dbGaP, already available
When available: With publication

Supporting Documents
Document types: None

Additional Information
Who can access the data: IRB approval needed; application to dbGaP
Types of analyses: Most purposes but not for studies of addiction or other sensitive phenotype
Mechanisms of data availability: After approval of proposal and with signed data access agreement
